# Supplementary material for: Investigating action topography in visual cortex and deep artificial neural networks
Source: Nat Commun. 2025 Dec 21;17:1094. doi: 10.1038/s41467-025-67855-6 (PMC12852736; doi:10.1038/s41467-025-67855-6)
Supplement: Supplementary file 1 — Supplementary Information [file 41467_2025_67855_MOESM1_ESM.pdf]

Supplementary Information

Investigating action topography in visual cortex and deep artificial neural networks

Davide Cortinovis, Nhut Truong, Hans Op de Beeck, Stefania Bracci

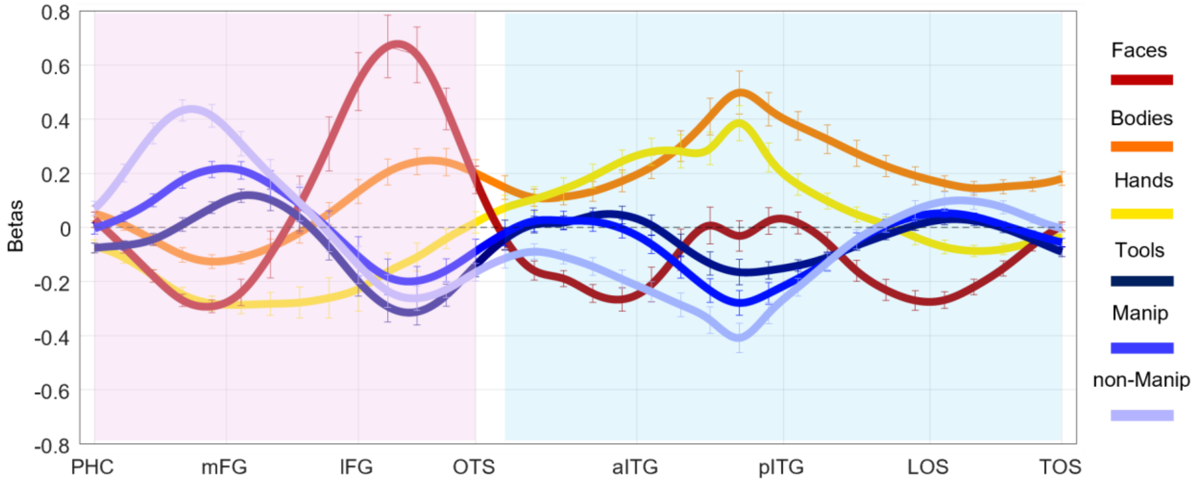

Figure S1. Right hemisphere vector-of-ROIs. The same procedure to generate the vector was followed as for the left hemisphere (see methods for details). The spheres along the vector cover an analogous portion of OTC as in the left hemisphere. Normalized activation (against the average of all categories) is plotted for each category as a function of the position of the vector along the cortex. The x-axis corresponds to each sphere along the vector, with labels for major anatomical landmarks; the y-axis corresponds to the normalized beta values. The vector was broadly divided into a ventral component (pink shade) and a lateral component (light blue shade). Contrary to the left hemisphere, no action-related organization can be observed in right lateral OTC. Error bars represent ± 1 SEM across participants (n = 18). Red = faces; orange = bodies; yellow = hands; dark blue = tools; blue = manipulable objects; light blue = non-manipulable objects. Source data are provided as a Source Data file.

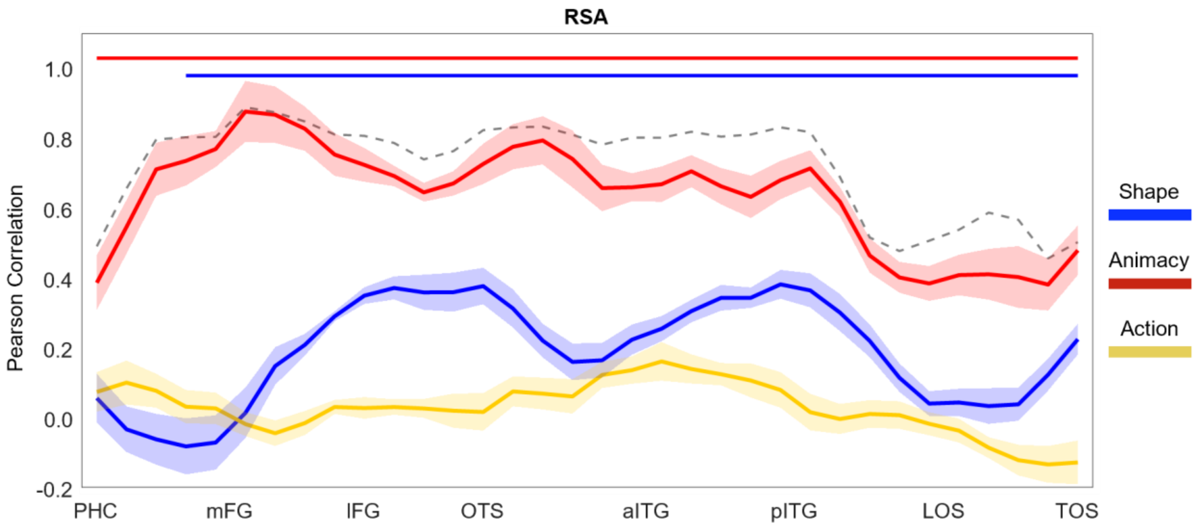

Figure S2. Object dimensions in the right hemisphere. Vector-of-ROIs RSA results. The dashed line represents lower bound of the noise ceiling. Two-sided one-sample t-tests were conducted, and horizontal lines indicate

statistical significance (vs. baseline) for each model ($p < .0014$ Bonferroni corrected for n = 34 comparisons; blue = shape; red = animacy; yellow = action). The shaded area around the line indicates ± 1 SEM across participants (n = 18). In no sphere of the vector there is a significant effect for the action model, indicating that animacy and – secondarily – shape dominates the object space in the right hemisphere. Source data are provided as a Source Data file.

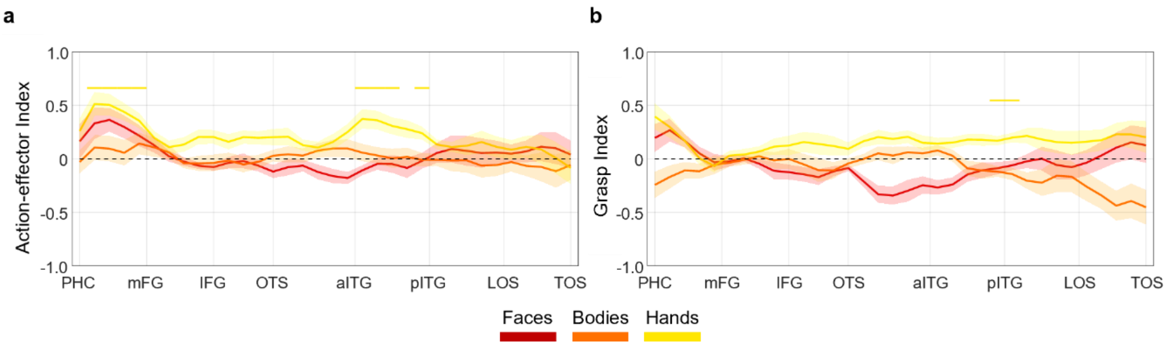

Figure S3. Index analysis. a) Vector-of-ROIs action-effector index. b) Vector-of-ROIs grasp index. Color-coded lines at the top of each plot indicate spheres along the vector where each index reached significance, corrected for the number of spheres (n = 34; $p = .0015$). Red = face indices; orange = body indices; yellow = hand indices. Some effects can be found in right LOTC and VOTC, indicating that, despite the lack of the general action information, hands and tools are moderately correlated with each other also in the right hemisphere. Source data are provided as a Source Data file.
