# Supplementary material for: Investigating action topography in visual cortex and deep artificial neural networks
Source: Nat Commun. 2025 Dec 21;17:1094. doi: 10.1038/s41467-025-67855-6 (PMC12852736; doi:10.1038/s41467-025-67855-6)
Supplement: Supplementary file 2 — Reporting Summary [file 41467_2025_67855_MOESM2_ESM.pdf]

nature portfolio

Corresponding author(s): Davide Cortinovis

Last updated by author(s): Nov 11, 2025

Reporting Summary

Nature Portfolio wishes to improve the reproducibility of the work that we publish. This form provides structure for consistency and transparency in reporting. For further information on Nature Portfolio policies, see our Editorial Policies and the Editorial Policy Checklist.

Statistics

For all statistical analyses, confirm that the following items are present in the figure legend, table legend, main text, or Methods section.

n/a	Confirmed	
☐	☒	The exact sample size (n) for each experimental group/condition, given as a discrete number and unit of measurement
☐	☒	A statement on whether measurements were taken from distinct samples or whether the same sample was measured repeatedly
☐	☒	The statistical test(s) used AND whether they are one- or two-sided<br>*Only common tests should be described solely by name; describe more complex techniques in the Methods section.*
☐	☒	A description of all covariates tested
☐	☒	A description of any assumptions or corrections, such as tests of normality and adjustment for multiple comparisons
☐	☒	A full description of the statistical parameters including central tendency (e.g. means) or other basic estimates (e.g. regression coefficient) AND variation (e.g. standard deviation) or associated estimates of uncertainty (e.g. confidence intervals)
☐	☒	For null hypothesis testing, the test statistic (e.g. F, t, r) with confidence intervals, effect sizes, degrees of freedom and P value noted<br>*Give P values as exact values whenever suitable.*
☒	☐	For Bayesian analysis, information on the choice of priors and Markov chain Monte Carlo settings
☒	☐	For hierarchical and complex designs, identification of the appropriate level for tests and full reporting of outcomes
☐	☒	Estimates of effect sizes (e.g. Cohen's d, Pearson's r), indicating how they were calculated

Our web collection on statistics for biologists contains articles on many of the points above.

Software and code

Policy information about availability of computer code

Data collection	Stimuli presentation and response collection was implemented using MATLAB R2021b (MathWorks: https://it.mathworks.com/products/matlab.html) and PsychToolbox-3 (http://psychtoolbox.org/) and custom-written MATLAB code.
Data analysis	All functional data preprocessing steps and first- and second-level analyses were conducted using SPM 12 (https://www.fil.ion.ucl.ac.uk/spm/software/spm12/). Subsequent analyses were executed with custom-written code in MATLAB (2021b) and publicly available at: https://github.com/DavideCortinovis/Action-topography-in-visual-cortex and https://osf.io/ctmbx/

For manuscripts utilizing custom algorithms or software that are central to the research but not yet described in published literature, software must be made available to editors and reviewers. We strongly encourage code deposition in a community repository (e.g. GitHub). See the Nature Portfolio guidelines for submitting code & software for further information.

Data

Policy information about availability of data

All manuscripts must include a data availability statement. This statement should provide the following information, where applicable:

- Accession codes, unique identifiers, or web links for publicly available datasets
- A description of any restrictions on data availability
- For clinical datasets or third party data, please ensure that the statement adheres to our policy

DATA AVAILABILITY

The following publicly available resources were used for this work: pretrained ResNet-action with Moments-in-Time and ResNet-object with ImageNet:

https://github.com/zhoubolei/moments_models,

TDANNs: https://github.com/neuroailab/TDANN. Beta values used for the fMRI analysis is available at: https://osf.io/ctmbx/

The single-subject and group-level fMRI data generated in this study is available through the Open Science Framework: https://osf.io/ctmbx/.

Research involving human participants, their data, or biological material

Policy information about studies with human participants or human data. See also policy information about sex, gender (identity/presentation), and sexual orientation and race, ethnicity and racism.

Reporting on sex and gender	Participants of both sexes (self-reported, M = 8, F = 11) were recruited for the fMRI experiment. Participants of both sexes (self-reported, M = 9, F = 13) were recruited for the behavioral experiment. Participants provided their sex/gender as part of a standard demographic questionnaire. However, sex/gender was not incorporated into the study, as we did not have hypotheses related to sex- or gender-based differences and the sample size was too small to support such analyses.
Reporting on race, ethnicity, or other socially relevant groupings	No race- or ethnicity-based exclusion, as the authors did not expect significant race or ethnicity differences.
Population characteristics	19 participants took part in the fMRI experiment (11 females, sex self-reported, mean age 25.6 years, standard deviation 6.06, unknown racial distribution), and 22 for the behavioral experiment (13 females, sex self-reported, mean age 23.3 years, SD = 1.96, unknown racial distribution). All participants were right-handed except one, all had normal or corrected-to-normal vision, and no history of neurological disorder.
Recruitment	Participants were recruited from the University of Trento community and participated in a single fMRI scanning session or in a single behavioral experiment session.<br>We do not expect biases in participant recruitment to meaningfully impact these results.
Ethics oversight	The Ethics Committee of the University of Trento approved the procedure.

Note that full information on the approval of the study protocol must also be provided in the manuscript.

Field-specific reporting

Please select the one below that is the best fit for your research. If you are not sure, read the appropriate sections before making your selection.

☒ Life sciences ☐ Behavioural & social sciences ☐ Ecological, evolutionary & environmental sciences

For a reference copy of the document with all sections, see nature.com/documents/nr-reporting-summary-flat.pdf

Life sciences study design

All studies must disclose on these points even when the disclosure is negative.

Sample size	The sample size was chosen based on previous studies employing similar data collection procedures. The number of participants in previous studies could range from 4-5 (with multiple sessions for each participant: Huth et al., 2012, Neuron; Kriegeskorte et al., 2008; Neuron) to 15-20 in a single session for each participant (Konkle & Oliva, 2012, Neuron, Konkle & Caramazza, 2021, Journal of Neuroscience, Yue et al., 2020, NeuroImage). A blocked design was used with a large number of block repetitions (n = 32 total for each category) resulting in highly reliable findings which replicate in each individual participant.
Data exclusions	Exclusion criteria were determined before the start of the data collection. Data for one participant was excluded due to excessive head motion (> 1 voxel, 3 mm in translation). Based on the same criteria, we further excluded two runs in two participants and one in a third participant (out of eight total for each participant).
Replication	The experiment was performed once, with data collected from 18 participants in a single fMRI session per participant. All analyses (univariate and multivariate) were independently performed across participants and yielded consistent results, confirming the robustness of the findings.
Randomization	No experiment groups were involved in the study: all conditions were tested within-individual.
Blinding	No group allocation performed.

Reporting for specific materials, systems and methods

We require information from authors about some types of materials, experimental systems and methods used in many studies. Here, indicate whether each material, system or method listed is relevant to your study. If you are not sure if a list item applies to your research, read the appropriate section before selecting a response.

Materials & experimental systems

n/a	Involved in the study
☒	☐ Antibodies
☒	☐ Eukaryotic cell lines
☒	☐ Palaeontology and archaeology
☒	☐ Animals and other organisms
☒	☐ Clinical data
☒	☐ Dual use research of concern
☒	☐ Plants

Methods

n/a	Involved in the study
☒	☐ ChIP-seq
☒	☐ Flow cytometry
☐	☒ MRI-based neuroimaging

Plants

Seed stocks

Report on the source of all seed stocks or other plant material used. If applicable, state the seed stock centre and catalogue number. If plant specimens were collected from the field, describe the collection location, date and sampling procedures.

Novel plant genotypes

Describe the methods by which all novel plant genotypes were produced. This includes those generated by transgenic approaches, gene editing, chemical/radiation-based mutagenesis and hybridization. For transgenic lines, describe the transformation method, the number of independent lines analyzed and the generation upon which experiments were performed. For gene-edited lines, describe the editor used, the endogenous sequence targeted for editing, the targeting guide RNA sequence (if applicable) and how the editor was applied.

Authentication

Describe any authentication procedures for each seed stock used or novel genotype generated. Describe any experiments used to assess the effect of a mutation and, where applicable, how potential secondary effects (e.g. second site T-DNA insertions, mosiacism, off-target gene editing) were examined.

Magnetic resonance imaging

Experimental design

Design type

Task; Blocked-design

Design specifications

In the fMRI experiment we collected 8 runs per participant. Each run lasted 400 sec (200 volumes). Each image was presented for 0.4 s, with an ISI of 0.266 s, in blocks of 8 s (i.e., 12 images per block). For each subject and for each run, a fully randomized sequence of all conditions was repeated 4 times, with a fixation block of 16 seconds at the beginning, in the middle (between sequences), and at the end of each run. 8 runs collected for each participant, resulting in 32 blocks per category.

Behavioral performance measures

A one-back task during the scanning was used to ensure participants' attention: specifically, participants were instructed to fixate their gaze on the fixation cross in the middle of the screen and press a button whenever the same image was repeated twice in a row within each block. The repeating image appeared once per block. Behavioral performance during the task was quantified by calculating response accuracy (mean = 93%, SD = 2.7%) and reaction times (RTs; mean = 0.6 s, SD = 0.02 s) for hits. Accuracy was defined as the proportion of correctly identified target stimuli, with responses considered correct if made within two trials following the targets, taking into account the fast presentation of the stimuli (0.4 s) and the reaction time of participants.

Acquisition

Imaging type(s)

functional and structural

Field strength

3T

Sequence & imaging parameters

The fMRI data was collected using a 3T Siemens scanner with a 64-channel head coil. MRI volumes were collected using echo planar (EPI) T2*-weighted sequence, with repetition time (TR) of 2 s, echo time (TE) of 28 ms, flip angle (FA) of 75°, and field of view of 220 mm. Each volume contained 50 axial slices, covering the whole brain, with matrix size 200 x 200 mm and 3x3x3 mm voxel size. Slices were acquired with a multiband (multi-slice) sequence. Anatomical images were acquired using the T1-weighted acquisition and MP-RAGE sequence, with a resolution of 1x1x1 mm.

Area of acquisition

Whole-brain

Diffusion MRI ☐ Used ☒ Not used

Preprocessing

Preprocessing software

The preprocessing was conducted using SPM12 and MATLAB (R2021b).

Normalization

Data were normalized to fit within MNI coordinates via SPM.

Normalization template

The data was normalized to a Montreal Neurological Institute's ICMB152 template with SPM.

Noise and artifact removal

To reduce noise and remove artifacts, the following standard preprocessing steps were applied to functional images: spatial realignment (to the first image) to correct for head motion; slice-timing correction; and, after coregistration and normalization, spatial smoothing with a Gaussian kernel of 4 mm FWHM to improve the signal-to-noise ratio.

Volume censoring

No volume censoring performed.

Statistical modeling & inference

Model type and settings

General Linear Model, random effect mass univariate analysis and multivariate analysis (RSA).

Effect(s) tested

Betas were extracted from the group-level GLM analysis and analysed in multiple ways, testing the functional selectivity of a broad region of ventral visual cortex via a procedure called vector-of-ROIs. Results were compared with analogous analysis in topographic artificial neural networks and further tested with multivariate analysis (RSA).

Specify type of analysis: ☐ Whole brain ☐ ROI-based ☒ Both

Anatomical location(s)

Univariate analysis were first conducted on the whole-brain. Further univariate and all multivariate analyses were conducted based on ROIs, which were identified with a procedure called "vector-of-ROIs", in which spheres are generated by fitting a spline connecting coordinates from previous studies.

Statistic type for inference

(See Eklund et al. 2016)

Cluster-level (FDR corrected at $p < .05$). This was applied only for the visualisations, ROIs selection and further statistical tests were independent from this threshold.

Correction

FDR correction was applied to the statistical tests conducted for the fMRI analysis. Multiple comparison correction via Bonferroni procedure applied for all other analysis (both univariate and multivariate). Permutations ($n = 10000$) were used for statistical analayses for computational models.

Models & analysis

n/a	Involved in the study
☒	☐ Functional and/or effective connectivity
☒	☐ Graph analysis
☒	☐ Multivariate modeling or predictive analysis
